# Effect of posture-control insoles on function in children with cerebral palsy: Randomized controlled clinical trial

**DOI:** 10.1186/1471-2474-13-193

**Published:** 2012-10-04

**Authors:** Hugo Pasini Neto, Luanda André Collange Grecco, Thaluanna CL Christovão, Luiz Alfredo Braun, Lilian Chrystiane Giannasi, Afonso Shiguemi Inoue Salgado, Renata Calhes Franco de Moura, Paulo de Tarso Camillo de Carvalho, João CF Corrêa, Luciana MM Sampaio, Manuela Galli, Claudia Santos Oliveira

**Affiliations:** 1 Post Graduate Program in Reabilitation Sciences, Universidade Nove de Julho, UNINOVE, Sao Paulo, Brazil; 2 Postdoctoral Fellowship of the Oral Bio pathology Post graduation Program- Unesp/Faculty of Dentistry, Sao Paulo, Brazil; 3 Therapist, Student in Doctor’s Program in Biomedical Engineering, Camilo Castelo Branco University, Sao Paulo, Brazil; 4 Associate Professor and director of “Luigi Divieti ”Motion analysis Lab, Dipartimento di Bioingegneria, Politecnico di Milano, Milan, Italy; 5 Motion analysis Lab, IRCCS San Raffaele Pisana, Tosinvest Sanità, Rome, Italy; 6 Integrated Laboratory of Human Movement Analysis, Universidade Nove de Julho - UNINOVE, São Paulo, SP, Brazil

**Keywords:** Cerebral palsy, Posture-control insoles, Ankle-foot orthosis, Electromyography, Gait, Stabilometry, Rehabilitation

## Abstract

**Introduction:**

Cerebral palsy (CP) is a posture and movement disorder and different therapeutic modalities, such as the use of braces, have sought to favor selective motor control and muscle coordination in such patients. The aim of the proposed study is to determine the effect of the combination of posture-control insoles and ankle-foot orthoses (AFOs) improving functional limitation in children with CP.

**Methods/Design:**

The sample will be composed of 24 children with CP between four and 12 years of age. After the signing of the statement of informed consent, the children will be randomly allocated to two groups: a control group using AFOs alone and an experimental group using both posture-control insoles and AFOs. Evaluations will be performed on five occasions: without any accessory (insoles or AFOs), immediately after, one month after, six months after and one year after AFOs or insole and AFOs use. The evaluation will involve the analysis of gait, static and functional balance, mobility and hypertonia. The three-dimensional assessment of gait will involve the eight-camera SMART-D SMART-D 140® system (BTS Engineering), two Kistler force plates (model 9286BA) and an eight-channel, wireless FREEEMG® electromyography (BTS Engineering). Static balance will be assessed using a Kistler force plate (model 9286BA). Clinical functional balance and mobility will be assessed using the Berg Balance Scale, Timed Up-and-Go Test and Six-Minute Walk Test. The posture-control insoles will be made of ethylene vinyl acetate, with thermal molding for fixation. The fixed orthoses will be made of polypropylene and attached to the ankle region (AFO). The results will be analyzed statistically, with the level significance set to 5% (p < 0.05).

**Trial Registration:**

Trial Registration Number: RBR6d342s (http://www.ensaiosclinicos.gov.br/news/)

## Introduction

Cerebral palsy (CP) is a permanent but not immutable posture and movement disorder resulting from a non-progressive cerebral disorder due to hereditary factors or events occurring during pregnancy, child birth, the neonatal period or in the first days of life, leading to limited motor activity and often accompanied by sensory, cognition, communication, perception and behavioral disorders. [1] The most current definition states that CP is a chronic, non-progressive disease with movement, posture and motor function disorders stemming from lesions or abnormalities in the immature brain [2].

Motor impairment is the major manifestation of CP, with consequent changes in bodily biomechanics. Moreover, children with CP may exhibit intellectual, visual and hearing disorders, which, when added to motor impairment and both task and environment restrictions, affect functional performance in a variety of different ways [3,4].

Neuromotor impairment in this disease involves different parts of the body, resulting in specific topographic classifications, such as quadriplegia, hemiplegia and diplegia [5]. However, children with CP are currently classified based on functionality, which encompasses the functions of the body, activities and social participation. The Gross Motor Function Classification System for Cerebral Palsy (GMFCS) [6] classifies children with CP based on age (0–2, 2–4, 4–6 and 6–12 years) and respective functional levels. Children classified as Level I can walk without restrictions, but tend to be limited in more advanced motor skills, whereas children classified as Level V are very limited in their ability to walk, even with a gait-assistance device [7]. The GMFCS is an extremely important tool for physical therapists who work with children with CP, as it allows the establishment of adequate therapeutic goals based on the patient’s age and motor level [7,8].

Functional mobility can also be assessed using the Berg Balance Scale and the Timed Up-and-Go Test. These scales allow a quantitative assessment of functional balance.

With regard to gait, a three-dimensional analysis allows a detailed evaluation of the kinetic, kinematic and electromyographic aspects of each phase of the gait cycle and is an important tool for the assessment of the results of clinical interventions in children with CP, who exhibit functional limitations due to excessive muscle weakness, abnormal joint kinetics and abnormal postural reactions [9].

Different therapeutic interventions seek to improve selective motor control and muscle coordination in these patients. The use of an orthosis (brace) is one such method, the aim of which, according to Lucarelli et al. (2007), is to improve the gait pattern [10]. Different types of orthosis may be prescribed, such as an ankle-foot orthosis (AFO), which assists in the alignment and quality of ambulation. This type of brace provides a reduction in plantar flexion of the ankle during initial contact with the ground, which leads to greater stability in the stance phase [11].

Similarly, the aim of posture-control insoles is to reorganize the tonus of muscle chains and influence body posture through correction reflexes. These insoles affect muscle proprioception, leading to changes in the ascending proprioceptive chains [12]. According to Gagey & Weber (2000), [13] the stimulation of specific regions of the sole of the foot leads to a change in postural tonus and a repositioning of the pelvis and muscle asymmetries along the spinal column. Postural reprogramming occurs when mechanoreceptors in the plantar region are activated by deformation of the skin due to the topographic relief of the support surface, as occurs with posture-control insoles [14].

The aim of the proposed study is to determine the effect of the combination of posture-control insoles and ankle-foot orthoses (AFOs) on functionality in children with CP. The hypothesis is that posture-control insoles lead to a change in sensitive afference, thereby stimulating a new postural reaction that favors better biomechanical alignment and allows greater efficiency in functional tasks, especially those related to locomotion and balance.

## Methods

### Type of study

A randomized, controlled, clinical trial will be carried out.

In compliance with Resolution 196/96 of the Brazilian National Health Council, the proposal was sent for the analysis of the Human Research Ethics Committee and received approval (August 8, 2011).

The children will participate on a volunteer basis and legal guardians will sign a statement of informed consent.

### Sample description and characterization

The sample size will be calculated based on the study carried out by Buckon et al. (2004)[15] with results on gait cadence in children with CP (GMFCS Levels I and II) with and without a fixed AFO. For an expected size effect of 17 steps/minute, with a standard deviation of 15 steps/minute and assuming an α risk of 0.05 and an 80% power, the sample was estimated at 12 children per group. Thus, the sample will be composed of 24 male and female children with CP aged four to 12 years**.**

The participants will be recruited and selected for eligibility based on the criteria listed below.

### Inclusion criteria

Diagnosis of CP; classification in Levels I and II of the GMFCS; and independent ambulation with no need for gait assistance devices (walker or crutches).

### Exclusion criteria

History of surgical procedures or application of phenol in previous 12 months; history of neurolytic blocks in previous six months; cognitive or visual impairment that might impede the performance of the tasks; ankle deformities not reducible to zero; and obesity [16].

### Sample composition

After fulfilling the eligibility criteria, the children will be randomly divided into two groups: 1) a control group that will make use of AFOs exclusively and 2) an experimental group that will make use of AFOs combined with posture-control insoles.

Children in therapy at rehabilitation centers will be recruited and instructed to maintain their normal therapy throughout the study. Randomization with involve a series of sealed opaque envelopes to ensure confidentiality. Each envelop will contain a card stipulating to which group the child will be allocated.

### Equipment

Body mass and stature will be determined using a duly calibrated mechanical scale (Welmy brand) with a 150-Kg capacity and precision of 0.1 Kg and stadiometer coupled to the scale with a precision of 0.1 cm.

Static balance will be evaluated using a force plate (Kistler, model 9286BA), which allows stabilometric analysis based on oscillations of the center of pressure. The acquisition frequency will be 400 Hz, captured by four piezoelectric sensors positioned at the ends of the platform, measuring 400/600 mm. The data will be recorded and interpreted using the SWAY software program designed by BTS Engineering, integrated to and synchronized with the SMART-D 140® system.

The SMART-D 140® system (BTS Engineering) will be used for the three-dimensional evaluation of gait, using eight cameras with an infrared-sensitive response and the 32-analog channel SMART-D INTEGRATED WORKSTATION®. The kinetic data will be collected using two force plates (Kistler, model 9286BA) for recording displacement from the center of pressure and the contact time between the foot and surface of the platform. An eight-channel, wireless-transmission electromyograph (FREEEMG® – BTS (Engineering) will also be used, with bipolar electrodes with a total gain of 2000 x and within a frequency of 1000HzHz. The impedance and common rejection mode will be >10^15^ Ω//0.2 pF and 60/10Hz 92 dB.

**Figure 1:**
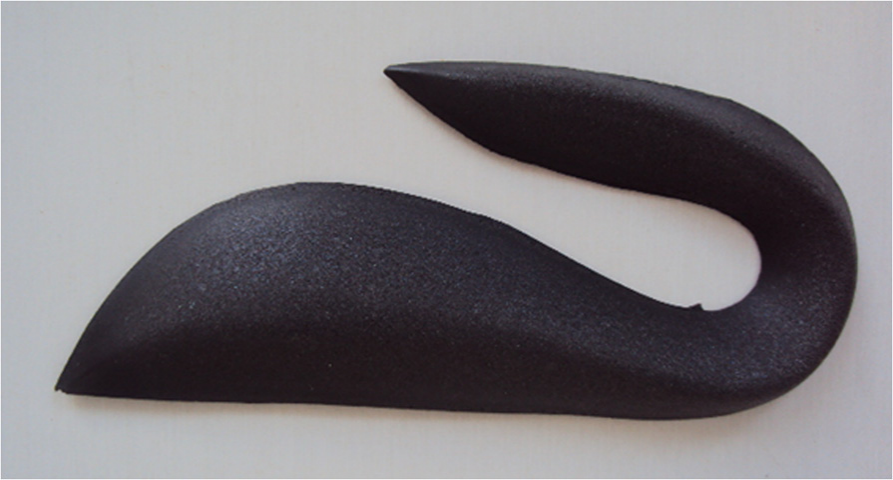
Representation of podal piece to be used in making of the posture-control insoles.

The posture-control insoles to be used by the children in the experimental group have surface, middle and deep portions. The surface portion is composed of fabric that covers the other portions and serves to absorb sweat and provide comfort. The middle portion is made of ethylene vinyl acetate (EVA) with a thickness of 3 mm. The lowest portion is made by material formed by a network of cotton fibers and resin with a thickness of 1 mm in which the podal pieces are located (bars, wedges and shims), made from EVA with respective thicknesses, densities and resilience, the aim of which is to stimulate the skin in predetermined regions and promote postural reprogramming [14]. In the study proposed, the pieces to be used will be the hard postural half moon, wedge and outer anti-rotator, with the aim of acting on the re-equilibrium of a common motor pattern (Figure 1).

**Figure 2:**
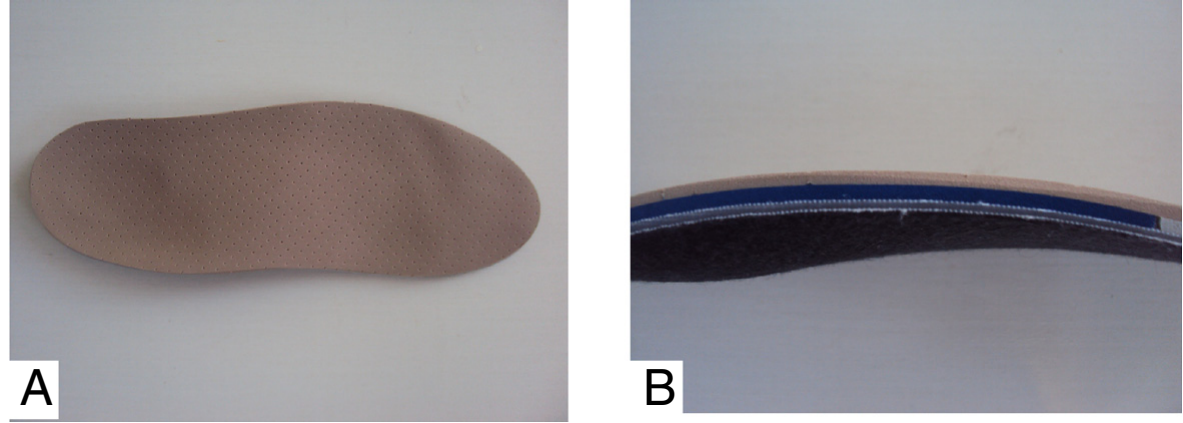
Representation of insoles after thermal molding; A – Front view; B- Side view, showing three portions.

After the different portions and the foot piece to be used are positioned, thermal molding of the insole will be performed for the fusion of the different sections and pieces (Figure 2). All material used for the confection of the insoles are from the brand name Podaly®.

### Instruments

The Gross Motor Function Classification System for Cerebral Palsy (GMFCS) will be used to classify the children based on the level of gross motor function [17]. This system classifies children between Levels I and V. Only children classified at Levels I and II will participate in the proposed study.

The motor growth curves [18] referring to GMFCS Levels I, II and III will be used as a complement to the classification of gross motor function. The curves have the scoring of the GMFM-66 [19,20] on the vertical axis and age on the horizontal axis for each GMFCS level. Using these curves, the child is functionally classified as being within the expected range, better than expected or poorer than expected.

The Berg Balance Scale will be used for the assessment of functional balance. This scale consists of 14 tasks that are similar to different activities of daily living. The items are scored on a five-point scale ranging from 0 (inability to perform task) to 4 (ability to perform task independently). The maximal score is 56 points. The point system is based on the time in which a position is maintained, the distance to which the upper limb is capable of reaching out in front of the body and the time needed to complete a task. The execution time is approximately 20 minutes. A chronometer, stool and chair are needed for the assessment. The evaluation is performed with the child dressed and making use of his/her habitual orthosis and/or gait assistance device [21,22].

The Timed Up-and-Go Test will be performed and distance will be measured using a metric tape. This fast, practical, easy-to-apply test is widely used for the assessment of functional mobility and dynamic balance. The test quantifies functional mobility through the time (in seconds) in which an individual performs the task (stand up from a standardized chair with back and arm supports, walk three meters, turn around, walk back to the chair and sit down again) [23,24].

The Six-Minute Walk Test will be performed. This test is a reliable measure for the assessment of physical fitness [25,26] and quantifies (in meters) functional mobility through the distance traveled walking in six minutes. This test will be performed based on the guidelines of the American Thoracic Society.

The modified Ashworth Scale will be used for the classification of hypertonia. This scale consists of Item 1+ and a five-point scoring system ranging from 0 (absence of tonic alteration) to 4. The classification of severity is related to range of motion at which increased resistance to rapid passive movement is detected [27].

### Procedures

The children selected (whose legal guardians agreed to their participation by signing a statement of informed consent) will be evaluated regarding their anthropometric data and GMFCS classification and randomly allocated to the experimental and control groups. The control group will make daily and constant use of the AFOs. The experimental group will make use of the posture-control insoles for six hours daily during the most active period of the day and will remain using the AFOs the rest of the day.

The evaluation process (before, immediately after, one month after, six months after and one year after insole use) will be performed under two different conditions. Under the first condition (evaluation 1), the children will not use an assistance device and under the second condition (evaluation 2), the children will use either the posture-control insoles or the AFOs, depending on the group to which they belong. Evaluation 1 and evaluation 2 will be performed by different examiners. Examiner 1 will not be aware of which group the children belong, thereby characterizing the investigation as a blind study.

The evaluations will be held on two non-consecutive days. The Berg Balance Scale, Timed Up-and-Go Test, Six-Minute Walk Test and modified Ashworth scale will be administered on the first day. The three-dimensional gait analysis and the static balance test on a pressure plate will be performed on the second day.

For the three-dimensional gait analysis, the equipment will be introduced to the children and the procedures will be explained. The children will be submitted to an initial practice gait exam to become familiarized with the procedure (no data will be collected on this training run). The children will wear bathing suits to facilitate the placement of the markers. The skin will be cleaned with alcohol for better attachment of the markers on the exact sites. The markers will be enveloped in adhesive tape lined with microscopic glass spheres and attached to a plastic base with double-sided adhesive tape. The markers will be attached to the children in the orthostatic position, as suggested by Davis et al. (1991), [28] on the following anatomic structures:

Pelvis: Three markers will be positioned on the anterior superior iliac spines (right and left) and one between the posterior superior iliac spines.

Thigh: One marker will be placed laterally to the greater trochanter. A second marker will be placed laterally to the lateral condyle of the femur. A third marker will be positioned on an appendicular line midway between the two previous points.

Leg: One marker will be placed laterally to the head of the fibula. A second marker will be placed laterally to the lateral malleolus. A third marker will be positioned on an appendicular line midway between the two previous points.

Foot: A marker will be placed laterally to the head of the fifth metatarsus. One adjunctive marker will be placed bilaterally on heel only for the standing acquisition, before the walking test.

The markers will be used as reference for the eight-camera SMART-D BTS^@^ system which will acquire the 3D coordinates (x,y,z) of each marker [29]. The set of markers will be used to estimate the position of the joint centers and calculate the three-dimensional kinematics of the pelvis, hips, knees and ankles [30]. This will be performed through the combination of coordinates, which will take the information obtained from the positioning of the markers into consideration [29].

For such, the children will walk on a track marked on the ground measuring 90 centimeters in width and four meters in length, with two force plates (model 9286A) positioned in the center. Upon stepping on the force plates while walking, the kinetic gait data will be collected and calculated using a video system (BTS, Milan, Italy) synchronized to the two force plates.

The electrical activity stemming from the activation of the rectus femoris, tibialis anterior and soleus muscles (on right and left leg) will be collected using a signal conditioner (FREEEMG®, BTS). For the placement of the six channels, the motor point of the muscles will be identified and the area will be cleaned with 70% alcohol to reduce impedance, based on the recommendations of the Surface Electromyography for the Non-Invasive Assessment of Muscles.^(31)^ All electromyographic data will digitized in 1000 frames/second using the BTS MYOLAB® software program. The kinematic and kinetic data will be collected simultaneously and managed using the BTS® system and Smart Capture® software program.

Static balance will be analyzed using a Kistler force plate (model 9286BA). The evaluation will be performed with the individuals in the orthostatic position on the force plate with no restriction regarding the foot base. Readings will be taken three times under two conditions (eyes open and eyes closed), with each reading lasting 30 seconds. A rest period will be respected between each application of the instrument and the child will be allowed to interrupt the evaluation to rest at any time.

### Flowchart

**Figure 3:**
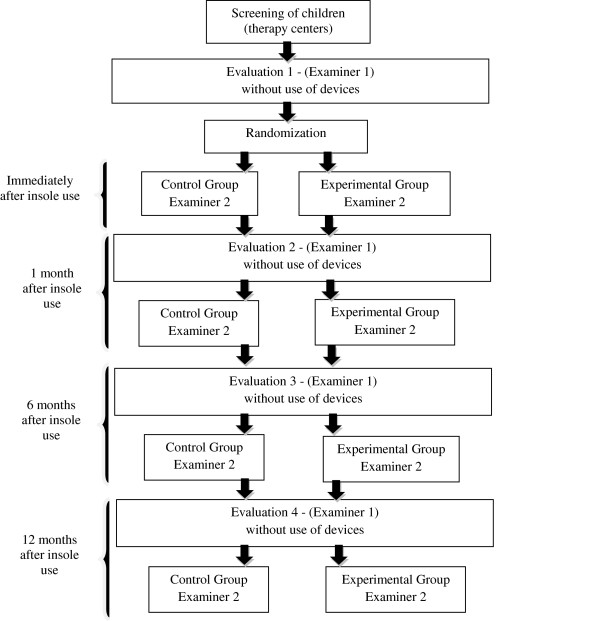
Flowchart of project.

The project will be carried out in accordance with the following flowchart (Figure 3).

### Statistical analysis

The Kolmogorov-Smirnov test will be used to test the data with regard to Gaussian distribution. Central tendency and dispersion measures will be expressed as mean and standard deviation values or median and inter-quartile interval when exhibiting parametric and non-parametric distribution, respectively. Either repeated-measure ANOVA or Friedman’s test will be used for the intra-group analysis and either one-way ANOVA or the Kruskal-Wallis test will be used for the inter-group analysis for data with parametric and non-parametric distribution, respectively. The data will be organized and tabulated using the Statistical Package for the Social Sciences (SPSS v.19.0). The level of significance will be set to 5% (p < 0.05).

## Competing interests

The authors declare that they have no competing interests.

## Authors’ contributions

All authors contributed to the conception and design of the study. CSO provided the idea for the study and established the hypothesis. HPN and LACG significantly contributed to drafting the manuscript. All authors read and approved the final manuscript.

## Pre-publication history

The pre-publication history for this paper can be accessed here:

http://www.biomedcentral.com/1471-2474/13/193/prepub
